# The role of L3 skeletal muscle index in the diagnosis and prognosis prediction of sarcopenia and malnutrition in cancer patients

**DOI:** 10.3389/fonc.2026.1759925

**Published:** 2026-03-04

**Authors:** Zhuo Chen, Deduo Xu, Xingyun Hou, Zhipeng Wang, Jinping Xiong, Mingying Geng, Jing Xu, Yanyan Gu

**Affiliations:** 1 Department of Oncology, Daping Hospital, Army Medical University, Chongqing, China; 2 Department of Pharmacy, Shanghai Changzheng Hospital, Second Military Medical University (Naval Medical University), Shanghai, China; 3 Department of Nutrition, Shanghai Changzheng Hospital, Second Military Medical University (Naval Medical University), Shanghai, China; 4 Department of Nephrology, Shanghai Changzheng Hospital, Second Military Medical University (Naval Medical University), Shanghai, China

**Keywords:** cancer, global leadership initiative on malnutrition criteria, malnutrition, reduced muscle mass, skeletal muscle index

## Abstract

**Objective:**

This study investigated the validity of the third lumbar skeletal muscle index (L3-SMI) as a marker of reduced muscle mass (RMM) in hospitalized cancer patients. It further evaluated the diagnostic accuracy of L3-SMI for malnutrition according to the Global Leadership Initiative on Malnutrition (GLIM) criteria and examined the relationship between L3-SMI and long-term survival in cancer patients.

**Methods:**

A retrospective analysis was conducted between April 2014 and November 2018 at Daping Hospital, affiliated with Army Medical University. A total of 284 cancer patients were included. Analytical methods used in this study comprised receiver operating characteristic (ROC) curve analysis, Kaplan-Meier survival analysis, Cox proportional hazards regression models, time-dependent ROC curves, and kappa statistics.

**Results:**

Using the GLIM criteria with L3-SMI, 99 patients (34.86%) were diagnosed with malnutrition. Cox regression analysis revealed that patients diagnosed with malnutrition based on L3-SMI had the highest hazard ratio (HR = 1.954, 95% CI = 1.291-2.958) for mortality. Kaplan-Meier survival analysis showed that malnourished patients had significantly poorer overall survival. The agreement between GLIM-SMI and the Patient-Generated Subjective Global Assessment (PG-SGA) in diagnosing malnutrition was moderate (Kappa = 0.550). Time-dependent ROC curve analysis demonstrated that the area under the curve (AUC) for GLIM-SMI was 0.617, 0.578, and 0.578 at 1-year, 3-year, and 5-year survival monitoring, respectively.

**Conclusion:**

The L3-SMI may be a useful alternative indicator for reduced muscle mass in hospitalized cancer patients. GLIM evaluated by incorporation of L3-SMI is independently associated with long-term survival outcomes in this patient population. The prognostic significance of GLIM-L3-SMI requires further validation in other cohorts with large sample size.

## Introduction

Cancer is a life-threatening disease characterized by uncontrolled cellular proliferation, in which tumor cells compete for nutrients to sustain rapid growth (1, 2). The relationship between cancer and nutritional status is critical, as malnutrition can severely affect patients’ quality of life, alter body composition, decrease functional capacity, and even negatively impact survival outcomes (3, 4). Advanced in treatment have improved the prognosis of cancer patients, enabling longer coexistence with the disease and underscoring the increasing importance of nutritional support. However, malnutrition is a complex condition influenced by multiple factors, including cancer treatments, psychological stress, inflammation, metabolic changes, increased energy expenditure, enhanced muscle catabolism, and reduced muscle mass (5–9). Early nutritional management, involving timely detection, regular assessment, and appropriate intervention, is crucial for both preventing and addressing malnutrition. Evaluating the effectiveness of these interventions has become necessary, despite historically being underestimated and overlooked (5, 10–12).

The diagnosis of malnutrition lacks a universally accepted global standard, as no single “gold standard” has been established to definitively determine malnutrition status. This underscores the need for appropriate nutritional assessments to identify malnutrition (4, 8, 13). In 2018, GLIM was introduced to integrate the best available evidence and expert opinion, with the aim of establishing a global consensus on malnutrition diagnosis in clinical settings (9). The GLIM criteria advocate a two-step approach: first, screening for nutritional risk, and second, diagnosing malnutrition based on specific criteria. These criteria consist of three phenotypic components (weight loss, low BMI, and RMM) and two etiologic components (reduced food intake or assimilation, and inflammation or disease burden). Among these, RMM is particularly challenging to assess due to the variety of methods available for its evaluation (9). Several imaging techniques, including bioelectrical impedance analysis (BIA), dual-energy X-ray absorptiometry (DXA), computed tomography (CT), and magnetic resonance imaging (MRI), have been suggested for accurately assessing RMM (9).

Computed tomography (CT) is widely regarded as a high-precision tool for assessing body composition and is routinely utilized in the field of oncology. With the rapid advancement of CT technology, it has gained the capability not only to differentiate between various tissue types but also to accurately quantify skeletal muscle (14–18). The third lumbar vertebra (L3) region has been validated as a reliable anatomical reference point for evaluating muscle mass using CT in cancer patients, as it serves as a strong predictor of overall muscle mass (19–23). The standardized cross-sectional area of muscle at L3 is referred to as the L3 skeletal muscle index (L3-SMI), and studies have demonstrated a significant correlation between L3-SMI and mortality in cancer patients (22, 24, 25). Furthermore, L3-SMI has been used in certain studies for the diagnosis of sarcopenia, often associated with aging (23, 26–28). Despite the promising results of CT-based muscle mass assessment, research that specifically utilizes CT-derived muscle measurements to meet the GLIM criteria for RMM remains limited (28–30).

This study aimed to evaluate the L3-SMI as a necessary and effective muscle mass biomarker for diagnosing malnutrition in hospitalized cancer patients under the GLIM criteria. The analysis was done by comparing the nutritional characteristics, quality of life (QOL), physical condition, clinical outcomes of patients, and the consistency of GLIM (using SMI) with the PG-SGA. Furthermore, the relationship between L3-SMI and long-term mortality was explored. To further validate the practicality of L3-SMI, the study utilized time-dependent ROC curves to compare the performance of L3-SMI with two other phenotypic standards (CC and MAMC) within the GLIM framework. This study hypothesized that RMM reflected by L3-SMI identified based on GLIM criteria is an effective tool for assessing malnutrition and predicting long-term survival to some extent in hospitalized cancer patients.

## 2 Materials and methods

### 2.1 Patient enrollment

This retrospective cohort study was conducted using data from the Oncology Department of Daping Hospital, Army Medical University, between April 2014 and November 2018. The inclusion criteria for the study were as follows: patients over 18 years of age, diagnosed with active cancer, hospitalized for more than 48 hours, and having undergone a preoperative abdominal CT scan. Patients were excluded if they were readmitted within 30 days or were in a terminal stage. After screening, a total of 284 patients diagnosed with gastrointestinal, respiratory, genitourinary, or other types of cancers were included in the study. Ethical approval was obtained from the Ethics Committee of Daping Hospital. The study was conducted in accordance with the ethical principles of the Declaration of Helsinki, under approval reference number (2018) No. 22-01.

### 2.2 Data acquisition

Within 48 hours of admission, trained dietitians or clinicians collected recent nutritional information from participants through face-to-face interviews. This data included body mass index (BMI), mid-arm circumference (MAC), and calf circumference (CC). Nutritional risk was assessed using the Nutritional Risk Screening 2002 (NRS2002), Karnofsky Performance Status (KPS) score, and the Patient-Generated Subjective Global Assessment (PG-SGA). Patients with a PG-SGA score of ≥9 were classified as malnourished (31, 32). Anthropometric measurements were conducted with precision, where body weight and height were measured to the nearest 0.1 kg and 0.1 cm, respectively, and BMI was calculated using the formula: (kg)/(m^2^). MAC and CC were measured with a soft, non-elastic tape, while triceps skinfold thickness (TSF) was assessed using a skinfold caliper. Mid-arm muscle circumference (MAMC) was calculated using the formula: MAMC = MAC - 3.14 × TSF. Grip strength was measured using an electronic handgrip dynamometer, with participants performing three maximal isometric contractions with their non-dominant hand, standing, and resting for one minute between trials. The highest value was recorded. Laboratory tests, including tumor staging and histological type, were based on fasting blood samples and hospital records during admission.

### 2.3 GLIM criteria

The criteria outlined by GLIM and their application for diagnosing malnutrition have been previously discussed (9). In summary, there are two main components: phenotypic and etiologic criteria. For assessing RMM, various measurements were used, such as the skeletal muscle index (SMI), left calf circumference (LC), and mid-arm muscle circumference (MAMC, non-dominant arm). To determine the presence of RMM, the 15th percentile (p15) for SMI, CC, and MAMC was separately calculated for each gender (33, 34). The etiologic criteria were based on the GLIM definition, and since all participants in the cohort were pathologically diagnosed with or undergoing treatment for cancer, the “disease burden” criterion was considered positive for all cases.

### 2.4 Evaluation of L3 skeletal muscle index

**Figure 1:**
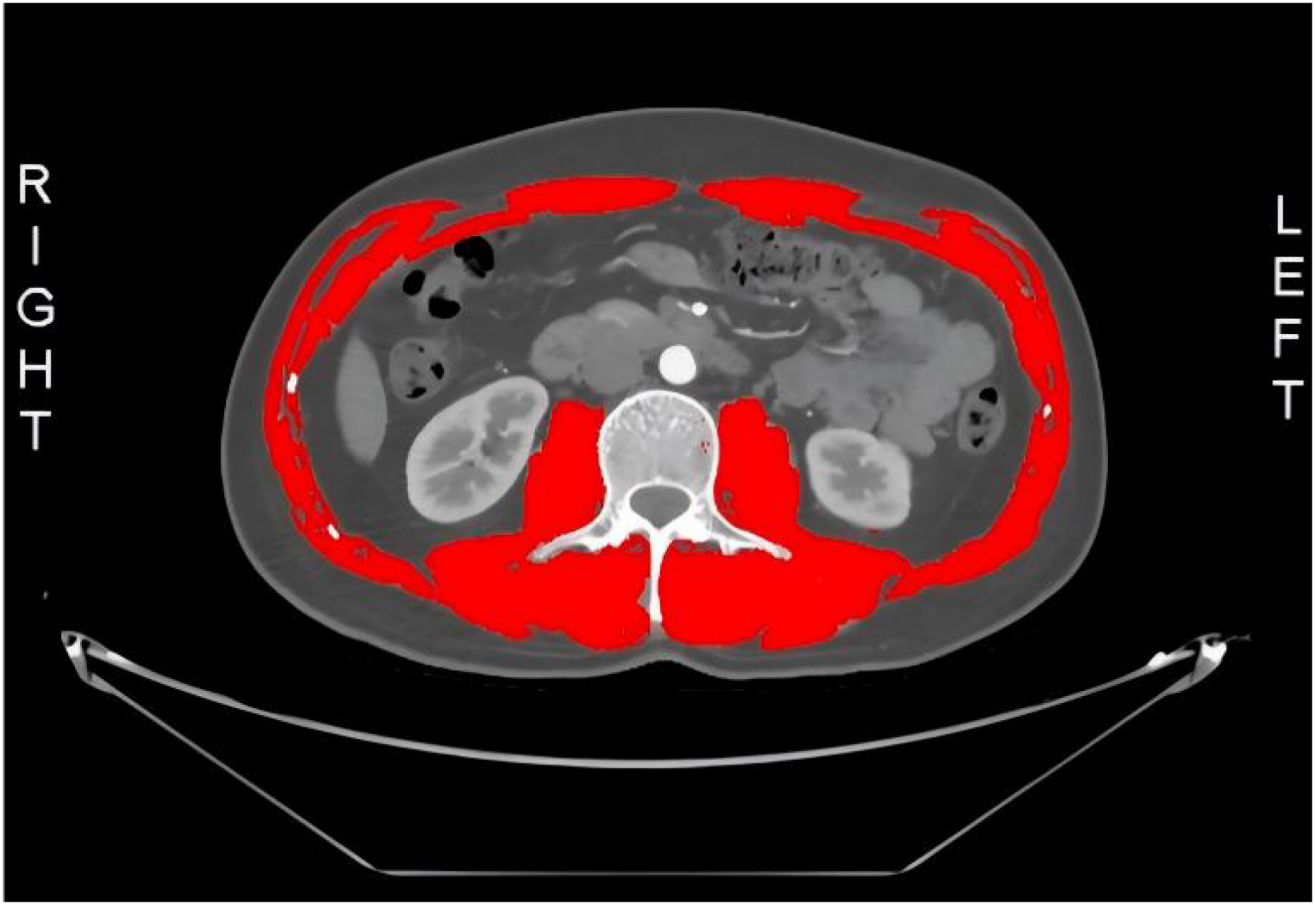
L3-SMI: third lumbar vertebra-skeletal muscle index.

Preoperative contrast-enhanced abdominal CT scans were performed for all patients, with acquisition parameters standardized across the cohort. Transverse CT images at the lower border of the third lumbar vertebra (L3) were chosen to assess muscle mass, following previously established methods (22, 35, 36). As shown in **Figure 1**, a trained researcher, blinded to the patients’ clinical and surgical data, processed the CT images using SliceOmatic V4.3 software (Tomovision, Montreal, QC, Canada). The software facilitates the identification of specific tissues, employing Hounsfield unit (HU) thresholds reported in prior studies. Skeletal muscles were distinguished from other tissues by applying an HU threshold range of −29 to +150. This included muscles such as the psoas major, erector spinae, quadratus lumborum, transversus abdominis, external and internal obliques, and rectus abdominis, with manual correction of tissue boundaries when necessary (27, 37, 38). The cross-sectional area of the L3 muscles was adjusted for height (cm^2^/m^2^) to calculate the L3 Skeletal Muscle Index (L3 SMI, cm^2^/m^2^) (24, 39).

### 2.5 Diagnostic consistency with PG-SGA

The Patient-Generated Subjective Global Assessment (PG-SGA) is a widely recognized nutritional assessment tool (11, 40). It was adapted by SGA, the PG-SGA is tailored specifically for cancer patients and is considered the gold standard for subjective nutritional evaluation in this population (12). In assessing the performance of the GLIM criteria for diagnosing malnutrition using skeletal muscle index (SMI), the PG-SGA was employed as the reference tool. The diagnostic agreement between GLIM and PG-SGA in identifying malnutrition was measured using the Kappa coefficient (Kappa), with interpretation of values as follows: 0.00–0.20 indicating slight agreement, 0.21–0.40 fair, 0.41–0.60 moderate, 0.61–0.80 substantial, and 0.81–1.00 representing almost perfect agreement (31, 32, 41).

### 2.6 Statistical analysis

Quantitative variables are expressed as mean ± standard deviation. The normality of continuous data was assessed using the Shapiro–Wilk test. To evaluate the homogeneity of variances, Levene’s test was applied. For data that did not meet the assumption of homogeneity of variances, comparisons were made using Welch’s analysis of variance (ANOVA). Categorical variables are presented as counts and percentages, with comparisons performed using the Chi-square test. For survival data, analysis was conducted using Kaplan-Meier survival curves, Cox proportional hazards regression, and time-dependent receiver operating characteristic (ROC) curves. All statistical tests were two-sided, and a P-value <0.05 was considered statistically significant. Statistical analyses were performed using R software (version 4.2.0).

## 3Results

### 3.1 Demographic and nutritional characteristics

The baseline characteristics of the study population are outlined in **Table 1**, with participants classified into malnourished and well-nourished groups. The study included a total of 71 women and 213 men, with an average age of 61 ± 10.77 years. Digestive system tumors were the most prevalent type of cancer, accounting for 59.51% of cases. Using a two-step process, 144 patients (50.70%) were identified as being at nutritional risk by NRS2002 screening, and 99 patients (34.86%) were subsequently diagnosed with malnutrition according to the GLIM criteria, using skeletal muscle index (SMI) as a marker.

**Table 1:** Demographic and nutritional characteristics of the study population stratified by the GLIM.

Characteristics	Overall (n=284)	Stratified by GLIM		
		Well-nourished (n=185)	Malnourished (n=99)	*P* value
Age, years, [mean (SD)]	60.95(10.77)	60.45(10.23)	61.87(11.72)	0.342
Sex, n (%)				0.943
male	213(75.00)	139(65.26)	74(34.74)	
female	71(25.00)	46(64.79)	25(35.21)	
Smoking, yes, n (%)	157(55.28)	106(57.30)	51(51.52)	0.350
Alcohol consumption, yes, n (%)	82(28.87)	53(28.65)	29(29.29)	0.909
Tea consumption, n (%)	67(23.59)	41(22.16)	26(26.26)	0.438
Primary tumor site, n (%)				0.030
Digestive system	169(59.51)	102(55.14)	67(67.68)	
Respiratory system	35(12.32)	28(15.14)	7(7.07)	
Urogenital system	28(9.86)	23(12.43)	5(5.05)	
others	54(19.01)	32(17.30)	20(20.20)	
TNM stage, n (%)				0.049
I	52(18.31)	40(21.62)	12(12.12)	
II	74(26.06)	50(27.03)	24(24.24)	
III	96(33.80)	57(30.81)	39(39.39)	
IV	62(21.83)	38(20.54)	24(24.24)	
NRS2002 score, continuous, mean ± SD	2.61(1.30)	1.88(0.92)	3.97(0.66)	<0.001
NRS2002 score, ≥ 3, n (%)	144(50.70)	45(24.32)	99(100.00)	<0.001
Weight loss within 6 months [mean(SD)],(%)	4.35(6.81)	1.48(4.39)	9.70(7.30)	<0.001
BMI, [mean (SD)],(kg/m^2)	22.29(3.73)	23.36(3.58)	20.28(3.14)	<0.001
HGS [mean (SD)], (Kg)	25.79(8.68)	27.14(8.34)	23.11(8.76)	<0.001
MAC[mean(SD)],(cm)	26.10(3.73)	27.09(2.80)	24.24(4.47)	<0.001
TSF[mean(SD)],(mm)	13.07(5.85)	13.98(5.94)	11.36(5.30)	<0.001
MAMC[mean(SD)],cm	22.01(3.32)	22.70(2.77)	20.70(3.87)	<0.001
CC[mean(SD)],cm	32.38(3.99)	33.41(2.92)	30.48(4.94)	<0.001
SMI[mean(SD)],(cm^2/m^2)	43.01(8.58)	45.18(8.37)	38.94(7.44)	<0.001
KPS score[mean (SD)]	90.88(10.58)	93.35(7.34)	86.26(13.75)	<0.001
Global quality of life by QLQ-C30[mean(SD)]	87.73(11.48)	90.40(7.93)	82.75(14.96)	<0.001
Neutrophils (×10^9/L) [mean (SD)]	5.46(9.61)	5.48(10.13)	5.43(8.60)	0.109
Lymphocytes (×10^9/L) [mean (SD)]	1.15(2.44)	1.14(3.05)	1.15(3.17)	0.042
NLR [mean (SD)]	4.76(13.14)	4.78(15.48)	4.72(6.99)	0.009
Platelet (×10^9/L) [mean (SD)]	199.96(76.08)	192.96(66.71)	213.04(89.97)	0.032
Hemoglobin (g/L) [mean (SD)]	126.63(21.44)	130.59(18.13)	119.22(25.01)	<0.001

GLIM, The Global Leadership Initiative on Malnutrition; BMI, body mass index; CC, calf circumference; MAMC: Mid-Arm Muscle Circumference; p15, the 15^th^ percentile; ^a^Males and females were evaluated separately; ^b^Requires one phenotypic criterion meeting this grade; ^c^percentile values of SMI (male, p15 = 37.55 cm^2^/m^2^; female, p15 = 31.24 cm^2^/m^2^); percentile values of calf circumference (male, p15 = 29.5 cm; female, p15 = 28 cm); percentile values of MAMC (male, p15 = 20.17 cm; female, p15 = 17.71 cm).

Significant differences were observed in the distribution of primary tumor sites and TNM stages between the malnourished and well-nourished groups (all P < 0.05). However, no significant differences were found in age, gender, smoking status, alcohol consumption, or tea drinking habit.

Various characteristics related to nutritional status, physical condition, and quality of life were compared between the two groups. Except for neutrophil levels, all measured variables showed significant differences (P < 0.05). More than half of the patients (158, 55.63%) were in advanced stages of cancer, with 33.80% in stage III and 21.83% in stage IV. Digestive system cancer remained the most common type (169 patients, 59.51%), followed by respiratory system cancer (35 patients, 12.32%), urogenital system cancer (28 patients, 9.86%), and other tumor types (54 patients, 19.01%). Body Mass Index (BMI) was significantly different between the groups, with well-nourished patients having an average BMI of 23.36 ± 3.58 kg/m^2^, compared to 20.28 ± 3.14 kg/m^2^ in malnourished patients (P < 0.001).

### 3.2 Consistency of GLIM method with PG-SGA diagnosis

The GLIM criteria demonstrated moderate agreement with the PG-SGA in identifying malnutrition within the study population. The diagnostic consistency of GLIM, in comparison to PG-SGA, showed a value of 0.889 (95% CI: 0.850–0.927), with a sensitivity of 0.788 and specificity of 0.832. The Kappa coefficient was 0.550, indicating moderate agreement (P < 0.001), as presented in **Table 2**.

**Table 2:** Analysis of the diagnostic agreement between the Patient-Generated Subjective Global Assessment (PG-SGA) and the GLIM-SMI method.

Category		GLIM	
		Normal	Malnutrition
PG-SGA	Normal	173	42
	Malnutrition	12	57

Accuracy (95%CI) = 0.889 (0.850, 0.927).

Sensitivity = 0.788, specificity = 0.832,

Kappa = 0.550, P < 0.001.

### 3.3 Kaplan–Meier survival analysis

**Figure 2:**
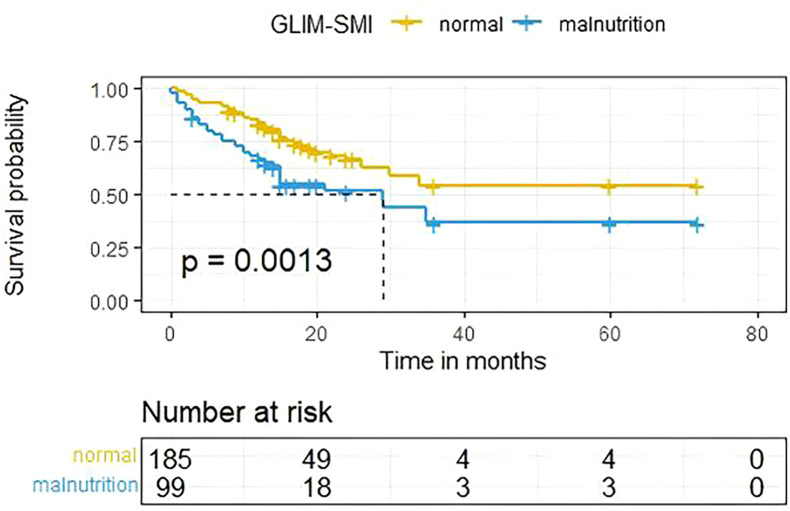
Results of the Kaplan-Meier survival analysis for cancer patients stratified by the GLIM-SMI.

As illustrated in **Figure 2**, Kaplan–Meier survival analysis showed that the overall survival (OS) of patients in the malnourished group was significantly poorer compared to the well-nourished group (P = 0.0013).

### 3.4 Relationship between subgroups and long-term survival

Based on univariate Cox regression analysis, lymphocyte count (P < 0.001) and hemoglobin level (P = 0.002) were significantly associated with mortality in cancer patients. Malnutrition, as diagnosed by the GLIM-SMI method (P = 0.002), GLIM-CC method (P = 0.012), and GLIM-MAMC method (P = 0.005), was significantly correlated with long-term survival in these patients. Specifically, patients identified as malnourished using GLIM criteria had worse overall survival (OS) in the intermediate and advanced TNM stages (Stage II: P = 0.049, Stage III: P = 0.008, Stage IV: P < 0.001). Additionally, the primary tumor location did not show a significant effect on long-term survival (**Table 3**).

**Table 3:** The results of the univariate Cox regression analysis of the cancer patients.

Variables	Univariate analysis	
	Hazard ratio (95% CI)	P value
Age	1.018 (0.998,1.039)	0.085
Sex		
male	Reference	
female	0.863(0.529,1.408)	0.555
Smoking	1.336(0.873,2.042)	0.182
Alcohol consumption		
No	Reference	
Yes	1.336(0.857,2.083)	0.200
Tea consumption		
no	Reference	
yes	1.350(0.858,2.123)	0.194
HGS	0.983(0.959,1.008)	0.174
Primary tumor site		
Digestive system	Reference	
Respiratory system	1.684(0.965,2.941)	0.067
Urogenital system	0.358(0.112,1.149)	0.084
others	1.298(0.776,2.171)	0.321
TNM stage		
I	Reference	
II	**2.499(1.003,6.227)**	**0.049**
III	**3.292(1.369,7.916)**	**0.008**
IV	**6.260(2.626,14.920)**	**<0.001**
Neutrophils	1.002(0.985,1.019)	0.848
Lymphocytes	**0.458(0.305,0.688)**	**<0.001**
NLR	1.003(0.992,1.014)	0.594
Platelet	1.001(0.998,1.004)	0.540
Hemoglobin	**0.985(0.976,0.994)**	**0.002**
GLIM-SMI		
Normal	Reference	
Malnourished	**1.954(1.291,2.958)**	**0.002**
GLIM-CC		
Normal	Reference	
Malnourished	**1.712(1.127,2.603)**	**0.012**
GLIM-MAMC		
Normal	Reference	
Malnourished	**1.809(1.192,2.746)**	**0.005**

HGS, Hand Grip Strength; GLIM, Global leadership Initiative on Malnutrition; NLR, Neutrophil-Lymphocyte Ratio.

Bold font indicates statistical significance (P<0.05). In univariate Cox regression, variables with bold hazard ratios (HR) are significant predictors of overall survival.

As shown in **Table 4**, multivariate Cox regression analysis, adjusted for clinical staging, lymphocyte count, and hemoglobin levels, indicated that the GLIM-SMI method—unlike the GLIM-CC and GLIM-MAMC methods—could be considered an independent negative prognostic factor for overall survival in malnourished patients (P = 0.04).

**Table 4:** Multivariable Cox regression analysis of the association between GLIM-diagnosed malnutrition and survival.

Characteristics	Number (%)	HR (95%CI)	P value
GLIM diagnosis(SMI)			
normal	185(65.14)	Reference	
malnourished	99(34.86)	**1.582(1.020,2.453)**	**0.040**
GLIM diagnosis(CC)			
normal	190(66.90)	Reference	
malnourished	94(33.10)	1.432(0.923,2.222)	0.109
GLIM diagnosis(MAMC)			
normal	190(66.90)	Reference	
malnourished	94(33.10)	1.520(0.981,2.354)	0.061

Using the SMI method, CC method and the MAMC method separately and both models were adjusted for clinical stage, lymphocytes and hemoglobin.

Bold font indicates statistical significance (P<0.05). In multivariate Cox regression, bold indicates that the variable remained an independent prognostic factor after adjustment for clinical stage, lymphocyte count, and hemoglobin level. An HR > 1 signifies an increased risk of mortality, while an HR < 1 signifies a decreased risk.

### 3.5 Time-dependent ROC curve for survival of cancer inpatients

**Figure 3:**
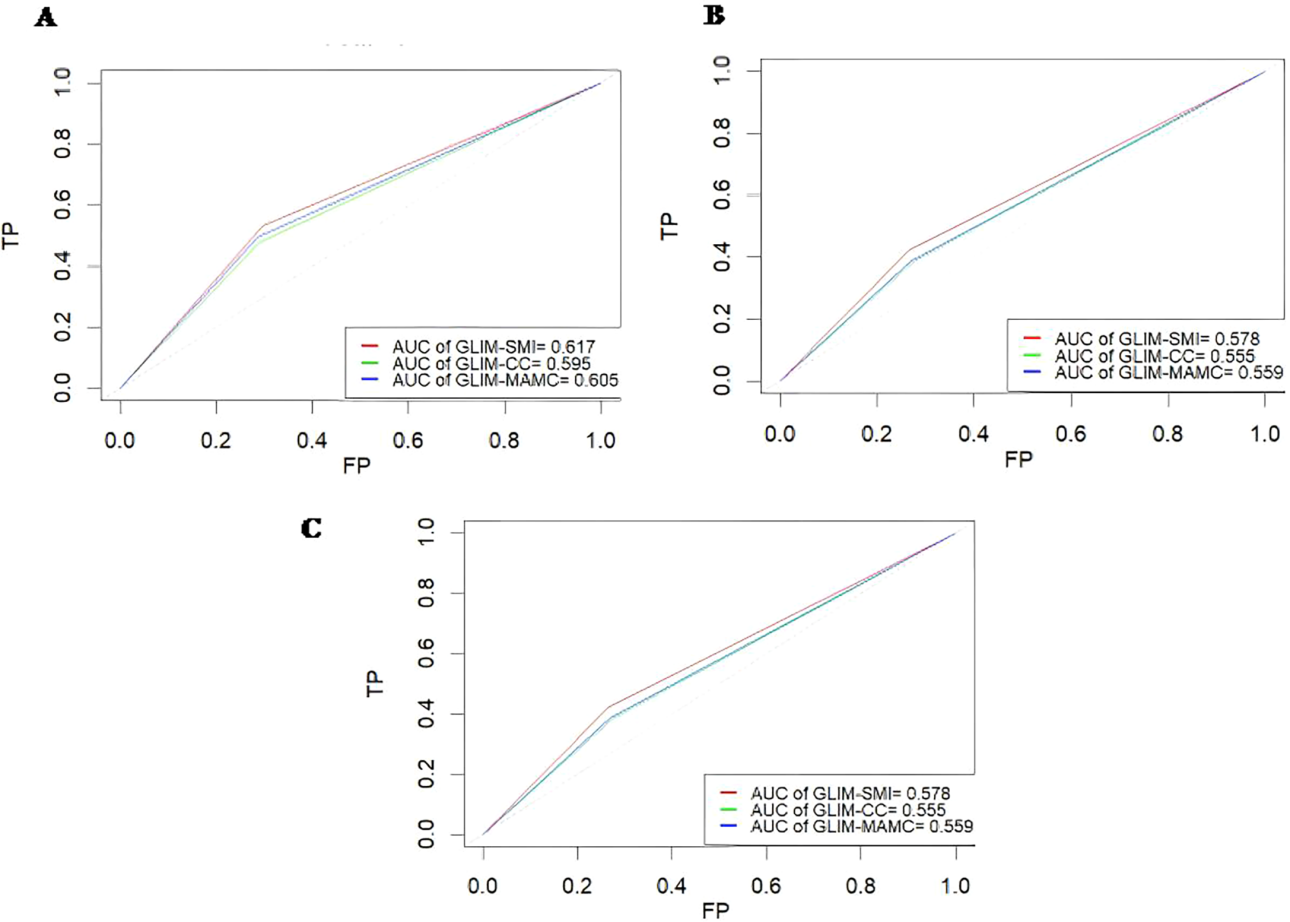
Time-Dependent ROC curves using different GLIM methods. **(A)** 1 year. **(B)** 3 years. **(C)** 5 years.

The predictive performance of L3-SMI was assessed using time-dependent ROC curves. The area under the curve (AUC) for GLIM-L3-SMI at 1-year, 3-year, and 5-year survival was 0.617, 0.578, and 0.578, respectively, indicating that GLIM-L3-SMI may be potential for survival monitoring. However, the predictive performance of GLIM-L3-SMI was just numerically higher than other metrics (GLIM-CC and GLIM-MAMC) (**Figures 3A–C**). These findings suggest that GLIM-L3-SMI could be a tool for monitoring survival.

## 4 Discussion

This study evaluated the use of L3-SMI as a marker for RMM under the GLIM criteria in diagnosing malnutrition in hospitalized cancer patients. Our findings indicate that L3-SMI may be an indicator of RMM for diagnosing malnutrition in cancer inpatients according to GLIM standards. Additionally, L3-SMI was significantly associated with long-term survival in these patients, suggesting its potential to predict prognosis to some extent.

In our cohort, the prevalence of malnutrition among cancer patients was 34.86%, which is lower than previously reported statistics. Studies conducted in Brazil, for example, have shown that approximately 40% to 80% of cancer patients and 45.3% of cancer inpatients experience malnutrition (42, 43). Significant statistical differences were observed in primary tumor locations and TNM staging between the malnourished and well-nourished groups. Compared to previous studies, tumor location typically varied between these two groups, although some studies found no significant differences in TNM staging (44). Kaplan-Meier survival analysis revealed that malnourished patients, as identified by GLIM-SMI, had poorer overall survival (OS), aligning with prior research (28). This further supports the utility of GLIM-SMI as a prognostic marker to differentiate malnourished patients. Additionally, the PG-SGA is widely recognized as the gold standard for subjective nutritional assessment in oncology. The diagnostic consistency between PG-SGA and GLIM-SMI yielded a Kappa value of 0.550 (P < 0.001), indicating fair diagnostic agreement. While this Kappa value is slightly lower than in previous studies, it does not diminish the significance of the findings (44, 45). Both univariable and multivariable Cox regression analyses confirmed a significant association between malnutrition, as determined by L3-SMI, and prognosis in cancer inpatients. Most studies, including ours, suggest that patients with RMM have significantly lower survival rates than those without RMM, irrespective of tumor stage or location, with the exception of survival rates associated with advanced tumor staging (3).

Certainly, there are limitations to our work. For example, time-dependent ROC curves indicate that the AUC value for GLIM-SMI is numerically slightly higher than for GLIM-MAMC or GLIM-CC, which may not directly prove that SMI is superior to MAMC and CC. We speculate that the sample size is a potential factor. Moreover, this study included patients with various types of tumors which is major source of heterogeneity. These two factors limited our ability to perform subgroup analyses based on tumor types. Additionally, some patients with multiple tumors may further have contributed to heterogeneity in the study population. This study employed SMI, CC, and MAMC with P15 to diagnose RMM, while some studies have also used P5 and P15, but P15 as a cutoff is more likely to yield false positives than P5. Therefore, the results should be interpreted with caution (33, 46). More importantly, the cut-off value for L3-SMI established in the present study should not be extrapolated to other populations without external validation. Moreover, we only screened patient data from a single center between April 2014 and November 2018; future studies should involve multi-center data collection over a broader time frame to further validate our findings.

Malnutrition severely impacts the quality of life of cancer patients and may lead to adverse clinical outcomes, including extended hospital stays, complications, and increased mortality rates. Muscle mass loss is significant evidence and a phenotypic criterion supporting malnutrition under GLIM standards. Previous statistics suggest that 38%-70% of cancer patients experience muscle mass loss (47). Cancer patients over the age of 40 can lose approximately 34% of muscle mass annually (48, 49). Extensive literature reports that cancer and its treatment can decrease patients’ muscle mass, especially in those undergoing longer treatment periods, where muscle loss persists (50, 51). L3-SMI can serve as an RMM marker for the diagnosis of malnutrition under GLIM standards. Therefore, L3-SMI can be incorporated into routine clinical examinations to detect and manage the health status of cancer inpatients and guide prognosis.

Certainly, routine CT scans, as conventional examinations for inpatients, possess excellent accuracy, and patient compliance with this examination is relatively high. This simple and practical examination method provides doctors with more information to achieve better diagnostic and prognostic outcomes. Additionally, to better identify and predict the health status of cancer patients or other patients, we can integrate other statistically significant findings from experiments, such as BMI, lymphocytes, NLR, hemoglobin, etc., to construct joint diagnostic and predictive methods. Besides identifying malnutrition and prognosis in cancer patients, further research may help doctors utilize L3-SMI or combined approaches to modify the diagnosis and prognosis of other catabolic diseases such as diabetes, nephrotic syndrome, tuberculosis, hyperthyroidism, and chronic atrophic gastritis.

## 5 Conclusion

In conclusion, L3-SMI can serve as a marker for RMM under GLIM standards for diagnosing malnutrition in hospitalized cancer patients. Furthermore, GLIM-defined malnutrition using L3-SMI is independently associated with and shows potential for predicting long-term survival in this patient population.

## Data Availability

The datasets presented in this study can be found in online repositories. The names of the repository/repositories and accession number(s) can be found in the article/supplementary material.
